# Moving Away from the Blame Culture: The Way Forward to Manage Medical Errors

**DOI:** 10.21315/mjms2024.31.6.10

**Published:** 2024-12-31

**Authors:** Aimi Nadia Mohd Yusof, Hazdalila Yais Haji Razali

**Affiliations:** Department of Medical Ethics and Law, Faculty of Medicine, Universiti Teknologi MARA, Sungai Buloh Campus, Sungai Buloh, Selangor, Malaysia

**Keywords:** blame culture, second victims, medical error, just culture

## Abstract

When a medical error occurs, the instinct to blame healthcare professionals may seems like a way to ensure they learn from their mistakes. However, in today’s healthcare landscape, the blame culture, coupled with the fear of litigation, proves detrimental to improving patient care. This culture fosters a reluctance among healthcare professionals to openly disclose mistakes, depriving them of valuable learning opportunities. These professionals, often referred to as second victims, deserve as much attention and support as the patients who are affected by the errors. Given that medical errors are inevitable, it becomes imperative to effectively manage the aftermath to ensure all parties involved are adequately supported and shielded from adverse consequences. This article delves into the ethical complexities of medical errors, advocating for a shift from a blame-centric culture to one that prioritises support for second victims. The aim of this article is to underscore the crucial importance of addressing medical errors within the healthcare sector by fostering an environment that promotes learning and growth post-error.

## Introduction

Unsafe care significantly threatens patient care and safety (1). In developing countries, it has been estimated that 4 in 100 patients die from unsafe care, which consists of medication, surgical, and diagnostic errors, as well as other unsafe practices (2). These instances of unsafe care are commonly referred to as medical errors, defined as unintended mistakes that can result in undesirable outcomes. However, it is important to note that in most cases involving medical errors, the errors are not caused by individual recklessness but are often due to a system failure (3).

Despite the possibility of system failures, there is often a tendency to identify and blame the individual who made the mistake rather than address broader systemic issues (4). Blame culture can be considered a significant obstacle to patient safety due to the fear of reporting and admission of fault in the organisation. It has also been suggested that this leads to the unacceptable rise of medical errors (5).

When medical errors occur, affected healthcare professionals seem to be more concerned with being blamed than with being punished (1). In fear of legal repercussions and being blamed for their mistakes, many healthcare professionals choose to remain silent instead of openly admitting their errors. In Malaysia, Hs and Rashid (6) found that only 10.1% of the doctors in their study would report any medical error. They believed that the low rate of disclosing medical errors might be due to a lack of policy on openness. Doctors are usually advised against disclosing medical errors to lawyers because of the risk of litigation (6). However, silencing and ignoring the patient’s right to know could eventually lead to aggravated damages once the case has been brought to court. This reluctance to discuss mistakes can result in further harm to patients if the underlying issues are not properly identified and addressed. Three key factors have been identified to hinder the practice of openness: i) blame culture; ii) fear of litigation; and iii) lack of policy supporting open discussion.

Blaming can be seen as a way to release the responsibility of others by attacking an individual. Blame culture is defined as a “set of norms and attitudes within an organisation characterised by an unwillingness to take risks or accept responsibility for mistakes because of a fear of criticism or management admonishment” (7). Blame culture leads to a punitive approach, deters the process of reporting errors, and can discourage healthcare professionals from coming forward to be honest and open about the mistakes that have occurred. They fear that if they are caught, they will be held individually responsible and punished for their conduct (2). This eventually limits any opportunities to improve the system and for the healthcare professionals involved to learn from their mistakes. If no data is available to initiate discussion to address errors, unsafe practices will continue to be unchecked (6). In dealing with medical errors, it is essential to emphasise the concept that allows the collaboration of justice, teamwork, and continual quality improvement (4). Acknowledging that anyone, including the most knowledgeable and experienced personnel, can make unintentional errors is essential.

The objective of this article is to explore the ethical ramifications of medical errors, with a focus on transitioning from a blame-oriented culture to one that offers support to second victims: healthcare providers affected by the mistakes. By cultivating an atmosphere that encourages learning and improvement after mistakes occur, the aim is to enhance awareness of the critical importance of effectively addressing medical errors within the healthcare sector. This article will only focus on managing medical errors with the support of second victims and does not cover the impact of errors on patients and their families in detail.

## The Problem with Medical Errors and the Impact on the Healthcare System

Medical errors, encompassing a range of issues from medication-related harm to patient falls, can inflict significant harm on patients and have profound personal, social, and economic impacts. Studies by Oura & Sajantila (8) and West (9) emphasise the severity and impact of these errors. Oura and Sajantila’s study showed a twofold increase in deaths due to medical adverse events among inpatients. West highlighted that preventable adverse events cause a substantial number of patient deaths annually in the United States, as revealed in the Institute of Medicine’s 1999 report. The World Health Organization (WHO) estimates that medication-related harm affects one in every 30 patients, with over a quarter of this harm being severe or life-threatening. Unsafe surgical care procedures lead to complications in up to 25% of patients, contributing to 1 million deaths annually across the world. Additionally, patient falls, which are the most frequent adverse events in hospitals, occur at a rate of 3 to 5 per 1,000 bed-days, with more than one-third resulting in injury.

Therefore, the WHO urgently calls for action by countries and global partners to reduce patient harm, as medical errors not only compromise clinical outcomes but also increase the financial burden on patients and the healthcare system (2, 8–10). Patient safety and quality of care are essential for delivering effective health services. Investing in improving patient safety can yield significant financial savings, as prevention costs are substantially lower than treating the consequences of medical errors (1).

The financial fallout resulting from medical errors is complex, involving costs linked to legal claims, settlements, increased insurance premiums, and investments in quality improvement initiatives. Institutions entangled in medical errors face various challenges, including legal and regulatory repercussions, such as potential lawsuits, fines, and disciplinary actions. Additionally, the aftermath includes damage to the reputation of the healthcare professionals and strained doctor-patient relationships. This impacts an institution’s standing in the healthcare community and dissuades potential patients from seeking services, potentially leading to a tangible decline in patient volume and revenue. It erodes public trust in healthcare organisations, leading to decreased patient satisfaction and loyalty.

Medical errors strain resources, disrupt patient care, and attract heightened regulatory scrutiny, necessitating system-wide improvements and initiatives that impact the entire healthcare ecosystem when addressing the root causes of the errors. These errors often require additional healthcare utilisation and interventions to rectify mistakes and manage ensuing complications. This translates to heightened hospital readmissions, additional surgeries, extended rehabilitation, and ongoing medical treatments, burdening patients and the healthcare system.

Despite the complications mentioned above, which are not exhaustive, there is a growing awareness of the need to reduce medical errors, especially those involving human error. However, despite efforts to minimise human error, healthcare professionals still make mistakes, underscoring the importance of understanding why these errors occur.

A study by Alexandrova-Karamanova et al. (11) in 2016 highlighted the impact of burnout on healthcare professionals and its potential role in medical errors. The study emphasised addressing burnout and related health behaviours to prevent medical errors. Arakawa et al. (12) conducted a study identifying predictors for medical errors among nurses, emphasising the importance of addressing working conditions and factors like illness, bodily pain, and emotional challenges to reduce error occurrence.

Burnout is not just an important factor prior to error. It can also occur after medical errors complicate its management. Following medical errors, doctors face significant emotional challenges, such as increased anxiety, loss of confidence, and other negative impacts (13). However, only a small percentage of doctors felt adequately supported by healthcare organisations in coping with errors-related stress.

Furthermore, healthcare professionals often experience feelings of guilt, shame, and self-doubt after such incidents, which will lead to burnout and increase the risk of future errors. It is crucial to recognise that in any medical error, not only the patient but also the institution and the healthcare workers are affected. However, there is often minimal attention given to healthcare workers affected by medical errors. They may have unintentionally found themselves in the wrong place at the wrong time, leading to their involvement in errors. They are often unheard and unappreciated, which can exacerbate self-blame and guilt, worsening burnout. Therefore, it is essential to support and care for these “second victims” affected by medical errors.

## Supporting Second Victims

Medical practice is commonly described as infallible with perfection, making it even worse for those affected by medical errors. It is essential to acknowledge that humans are fallible, and errors will occur even for the most well-intentioned, intelligent, and skilled healthcare professionals (13). Most of the time, if an error is identified, management of the error focuses only on the patient without properly acknowledging the need to address the well-being of healthcare practitioners impacted by the event. The welfare of the healthcare practitioners involved in the error is often neglected, allowing them to experience all the negative consequences that could impair not just their safety, especially emotionally, but also the safety of the patients. They are usually referred to as second victims. Second victims are healthcare workers who are affected by an unexpected negative patient outcome, such as a medical error, and consequently experience trauma from the incident.

Second victims need protection and support to continue working in a culturally positive environment. Once an error happens, prompt support, such as counselling and leave from work, would be needed to allow them to cope with the stress. Blaming them worsens the situation and hinders necessary improvements to manage the post-event. As stated earlier, when an error occurs, it is most likely due to a system failure rather than an individual mistake. Therefore, it is unfair to blame a single person for carrying the responsibility of committing the error alone.

Without adequate support, the second victim will have decreased quality of life, depression, and burnout (13), leading to unsafe patient care. On the other hand, with adequate support, healthcare practitioners working in a supportive environment could move forward to learn from mistakes and improve the system for a better healthcare environment. Eventually, this culture of learning leads to a better error management system that allows openness without fear of being blamed.

## Moving Away from Blame Culture to the Culture of Learning

Moving away from a blame culture to a culture of learning is crucial to ensuring the well-being of second victims. Promoting a blame-free culture is among the most effective strategies for managing medical errors (14), serving as a strong motivator for fostering a culture of learning and improvement. However, there needs to be sufficient support to address the error.

The culture of blaming and shaming inhibits system improvements and the future avoidance of medical errors. Furthermore, institutionalising the culture of blame leads to a culture of defensiveness (15). Allowing defensive medicine to continue will only impair patient care and deter doctors from speaking openly and honestly about learning from their mistakes. This process is a malignant experience that does not create any learning efforts from the incident. Implementing interventions, such as prioritising system improvements, refraining from individual blame, cultivating supportive training and work environments, and promoting personal wellness, can help mitigate the adverse effects of medical mistakes (13).

It is not accurate to say that making mistakes is acceptable. However, it is crucial to consider the intentions and attitudes of healthcare workers when errors occur. While it may seem unjust to discipline individuals who made honest errors, those identified as reckless should face consequences to safeguard patient safety (16). In instances of intentional mistakes, appropriate disciplinary measures should be taken to address the need for accountability. Balancing personal accountability and a no-blame strategy to manage errors may be challenging, but it is vital to move away from blame while simultaneously encouraging a sense of responsibility to ensure that those involved learn from mistakes and will avoid future errors. Careful considerations need to occur to avoid failing to identify those repeatedly making medical mistakes in a no-blame culture. There must be a balance between blamelessness and punishment to ensure a smooth process for reporting errors and create a positive cultural environment that leads to a just culture. In order to achieve this, strong support from the hospital management is needed.

## Way Forward to Manage Medical Errors

Transitioning from a blame-oriented culture involves prioritising initiatives that enhance service, minimise errors, and distribute responsibility throughout the healthcare system. This shift indirectly benefits healthcare staff. By identifying errors within institutions, collaborative efforts can be made to establish a strong safety net for both patients and healthcare workers without assigning blame.

The path to managing medical errors lies in fostering a culture of learning rather than giving blame, providing support, and providing mechanisms for doctors to disclose their mistakes. In any future instances of medical errors, healthcare professionals should prioritise identifying the error, understanding why it has occurred, rectifying it, addressing patient care following the error, including disclosing the error to patients and supporting the second victims. Some of these processes should start simultaneously to ensure a holistic approach from the beginning until the end.

Effective medical error management relies on implementing a reporting and learning system that encourages the reporting of errors, near misses, and adverse events. The primary goals of error reporting systems are learning, sharing, and exchanging information from past failures to enhance patient safety. Institutions leverage reporting programmes as valuable tools for identifying system-based issues in medication management, primarily through voluntary incident reporting mechanisms. However, the frequency of reporting varies among institutions, often due to barriers such as uncertainties, heightened burdens on healthcare providers, organisational factors, blame culture, and fear-induced concerns (17, 18).

Despite healthcare professionals’ familiarity with the Medication Error Reporting System and their encounters with medication errors in clinical settings, significant barriers to reporting were identified, attributed mainly to the demanding nature of the work environment (19). While numerous studies emphasise the obstacles to reporting, practical strategies can facilitate reporting and enhance patients’ safety. These strategies include implementing straightforward reporting forms, providing comprehensive training, offering anonymous reporting options, establishing feedback mechanisms, recognising error severity, and fostering supportive work environments. Embracing these measures can cultivate an atmosphere conducive to open dialogue and efficient error handling, promoting transparency, accountability, and continuous improvement in healthcare delivery.

It is imperative that those in the high positions of an institution involved in the decision-making process are made aware of their role in responding to medical errors and are adequately trained to execute this role. They are the core of making improvements to the environment of an institution. They need to be able to collaborate well with all the stakeholders involved in an error without being prejudiced and leaning towards a just culture. Research shows leadership plays a critical role in reducing medical errors through organisational culture. He highlighted that effective leadership is crucial for reducing errors and improving patient safety. Strategic leadership can greatly influence safety outcomes, particularly by shaping organisational culture to prioritise safety (7).

**Figure 1:**
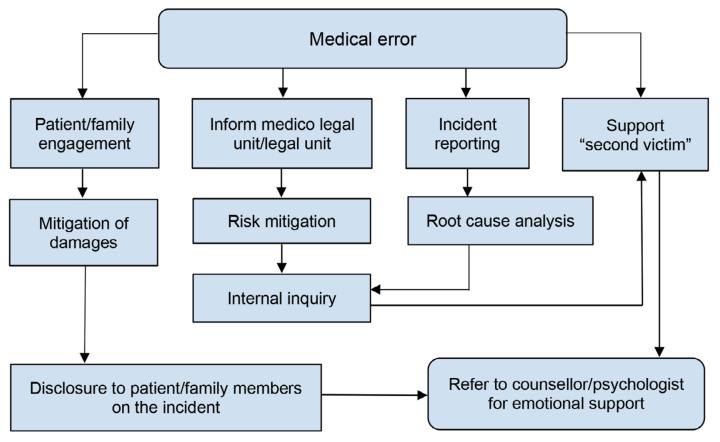
The way forward in managing medical errors

One crucial step forward in managing medical errors while prioritising the well-being of the second victim is implementing a policy that promotes a just culture, as stated above. A just culture focuses more on identifying potential risks and preventing harm to patients than on solely punishing individuals for human error. It is essential to establish clear expectations for error management so that all employees understand what to anticipate if they make a mistake. Consistently applying a review process for behaviour and actions following each error is imperative to ensure that no important stakeholders or steps are overlooked. These efforts to manage medical errors within a just culture are further illustrated in Figure 1.

In an adverse event, early involvement of a medicolegal adviser and a counsellor or psychologist is important to assist in the mitigation of risks. Upon discovery of a medical error, the first step involves promptly reporting it while engaging with the patient simultaneously. Subsequently, a root cause analysis (RCA) should be conducted at the management level to determine whether the error resulted from human or systemic factors. Through the RCA process, healthcare institutions aim to identify all contributing factors leading to an adverse event. Essentially, RCA investigations continually probe why a medical error occurred until all underlying deficiencies in the system are uncovered. RCA emphasises shortcomings in system-level processes rather than individual actions. Following a sentinel event, a designated RCA team must be assembled to review and pinpoint necessary systemic changes that can enhance performance and reduce the likelihood of a recurrence. For accreditation purposes, healthcare institutions are required by bodies such as the Joint Commission International (JCI) and the Malaysian Society for Quality in Health (MSQH) in Malaysia to have a comprehensive process for systematically analysing sentinel events.

With regard to the patient’s management, the responsible team should assess their understanding, acceptance, and expectations. The duty of candour of medical professionals necessitates those patients be informed of the event as soon as it is feasible; concealing the truth is considered unethical. However, the process of disclosure is a delicate matter that needs careful attention to ensure the process can mitigate risks rather than escalate them. The responsible team should also establish rapport with the patient and family members to demonstrate empathy, compassion, and accountability. It is crucial to convey to the patient that the event was unintentional, expressing empathy and remorse for the incident as well as emphasising that there will be no future occurrences.

In making sure that the quality improvement process takes place, training of doctors is important to ensure they are well prepared to face the challenges of medical errors, especially the senior consultants who are expected to manage medical errors better. They need to be aware of the negative impact of blame culture and move towards a learning culture for continual quality improvement. A well-trained system involving all stakeholders in managing medical errors could potentially reduce the risks of medical litigation. At the same time, the well-being of all affected by the error could also be protected.

## Conclusion

Recognising the notion of second victims, healthcare professionals affected by medical errors need to be in a supportive environment that promotes learning and growth. Through the provision of sufficient support and resources, healthcare organisations can alleviate the adverse effects of medical errors on the well-being of healthcare professionals and, thereby, on patient care. Efforts to transition away from a blame culture must be complemented by systemic changes aimed at preventing errors and enhancing patient safety. Moving forward, it is imperative for healthcare institutions, governmental bodies, and legal entities to collaborate in fostering a culture that values learning, system improvement, and patient safety. By embracing these principles, healthcare systems can effectively manage medical errors, promote a culture of trust and accountability, and ultimately enhance the quality of care provided to patients worldwide.
